# The Role of Stress and Perceived Social Support in the Association Between Perceived Discrimination and Mental Health Among Migrant Domestic Workers in Hong Kong

**DOI:** 10.1007/s10903-025-01694-x

**Published:** 2025-05-20

**Authors:** Timothy S. Sumerlin, Jean H. Kim, Roger Y. Chung

**Affiliations:** 1 Jockey Club School of Public Health and Primary Care, The Chinese University of Hong Kong, Sha Tin, Hong Kong SAR China; 2 Center for Global Health Equity, New York University Shanghai, Shanghai, China; 3 Centre for Bioethics, The Chinese University of Hong Kong, Sha Tin, Hong Kong SAR China; 4 Institute of Health Equity, The Chinese University of Hong Kong, Sha Tin, Hong Kong SAR China

**Keywords:** Perceived discrimination, Stress, Perceived social support, Anxiety, Depression, Migrant domestic worker

## Abstract

International migrant workers often face discrimination and poor work conditions that may negatively affect their mental health. Female migrant domestic workers (MDW), who often must reside in their place of work, may face additional daily stressors and discrimination compared with other international migrant workers. To address the stresses from discrimination and other challenges faced by MDWs, use of social support networks are often encouraged to improve mental well-being. This study seeks to examine the mediating effect of stress between perceived discrimination and self-reported mental health and the moderating role of perceived social support. An online cross-sectional survey was conducted during August 2020 – August 2021 among female MDWs in Hong Kong, China (*n* = 1965). In addition to assessing the association between perceived discrimination with anxiety (GAD-7) and depression (PHQ-9), a moderated mediation analysis examined the mediating role of stress and moderating role of perceived social support. Of respondents, 60.4% experienced at least occasional discrimination. Higher levels of perceived discrimination were significantly directly associated with both increased anxiety and depression (*p* < 0.05). Stress significantly mediated this association. Perceived social support significantly moderated the mediation pathway but did not moderate the direct effect of perceived discrimination on mental health. Increasing social support channels may alleviate the effects of increased stress from perceived discrimination on MDWs’ mental health. Policy-level campaigns to reduce discrimination against migrant workers should be considered.

**Supplementary Information:**

The online version contains supplementary material available at 10.1007/s10903-025-01694-x.

## Background

The mental health toll of perceived discrimination among minority and migrant populations has been an important area of research. Perceived discrimination, the subjective experience of unfair treatment due to a socially defined characteristic, can perpetuate inequalities between socially constructed groups [1–3]. Perceived discrimination has been linked to increased risk of psychiatric symptoms like anxiety and depression across different minority groups [1, 4–7].

International migrant workers, numbering 167.7 million globally in 2022, often face poor working conditions and social isolation, both of which contribute to poor mental health [8–13]. Among them, migrant domestic workers (MDW) – numbered 8.5 million globally in 2013 (most recent available data), with likely higher numbers today [13, 14] – face unique challenges due to their employment conditions. MDWs, who provide essential domestic labor including cleaning, cooking, and caregiving, often live in their employer’s home, a common policy requirement which may worsen employment conditions and mental health [15–18].

The *kafala* sponsorship system in the Middle East and similar programs in East Asia tie MDWs’ legal residency to their employer, which limits MDW autonomy and contributes to human rights violations, discrimination, and increased risk of poor health [18–24]. Perceived discrimination based on MDWs’ employment status and their employment conditions have been reported in East Asia, the Middle East, and Africa [17, 25–29]. In qualitative literature, MDWs attributed workplace discrimination to perceived employer-employee power imbalances, excessive working hours, distrust by the employer, and dehumanizing treatment [25, 30]. Limited literature from East Asia demonstrates associations between perceived discrimination and mental health among MDWs [17, 31]. However, mediating and moderating factors within the pathway of this association are not well studied.

### Theoretical/Conceptual Framework

Perceived discrimination based on occupational status, which has been commonly reported among MDWs, is likely to cause heightened stress. However, while high stress from working often long hours and in poor conditions is known among MDWs, its relationship with perceived discrimination has not been explored in this population [9, 18, 32–34]. Discrimination may trigger stress responses like increased vigilance and anticipation of further discrimination, worsening mental health [35, 36]. In other migrant groups, stress has mediated the association between discrimination and mental health [37–39]. However, MDWs lack of financial and legal security compared with other migrant groups suggests they may face even worse distress from perceived discrimination [40], warranting further study.

Social support is an important resource for MDWs as they are separated from left-behind family and friends. As theorized, perceived social support, the cognitive appraisal of being supported and connected to others [41], may buffer the effects of discrimination and stress on mental health [42–44]. MDWs reporting strong social support networks have exhibited better resilience and mental health [45–47]. However, social support’s buffering effect on perceived discrimination and consequent stress on mental health among MDWs has not been examined, and mixed results of this effect have been found among other minority and migrant groups [43, 44].

**Fig. 1:**
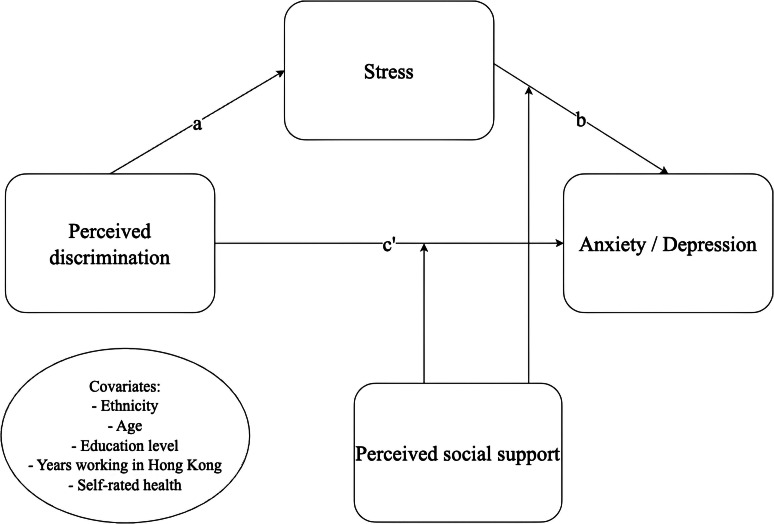
Conceptual framework. Stress was measured using a validated single-item measure for stress [61]. Anxiety and depression were measured using the Generalized Anxiety Disorder – 7 [65] and Patient Health Questionnaire – 9 [66] scales, respectively. All items were self-reported

Since the 1970s, Hong Kong has recruited women, primarily from Southeast Asia, to work as MDWs, with 356,231 female MDWs employed in 2022 [48]. In Hong Kong, where living spaces are among the smallest in the world [49], MDWs are legally required to live in their employer’s residence and have no limit on weekly working hours [50]. These conditions contribute to perceived discrimination and increase exposure to other forms of discrimination [25, 30]. In addition to reported occupation-based perceived discrimination [25, 30, 51], there is evidence of discriminatory attitudes towards MDWs by employers online and through the local Hong Kong press [27, 52, 53]. These attitudes may stem from perceptions of MDWs as socially inferior, migrants, and ‘other,’ and their employment conditions as under-regulated [53]. While the health effects of discrimination on migrant groups are well-documented in Western and Gulf countries, research in East Asia is limited [29, 54–58]. While systemic causes of discrimination may require long-term policy reforms, identifying targetable and modifiable mechanisms related to discrimination’s effect on mental health is critical for MDWs’ immediate well-being. Therefore, this study seeks to (1) assess stress as a mediator between perceived discrimination and anxiety/depression, and (2) evaluate the buffering effect of perceived social support (Fig. 1).

## Methods

### Participants and Data Collection

Data collection took place in Hong Kong using a cross-sectional online self-administered survey between August 2020 and August 2021. Female MDWs from either the Philippines or Indonesia who had worked as a MDW in Hong Kong for at least one year were eligible to take part in the survey. Two sampling approaches were used to complete the online survey: (1) a multi-stage cluster random sampling, based on a systematic random approach by reaching MDWs on their day off while gathering in public areas [59] (37% of sample), and (2) non-probability sampling online through social media platforms (i.e. Facebook and WhatsApp) (63% of sample). The survey was available in both English and Indonesian and underwent pilot testing for understanding and cultural validation. Informed consent was obtained by all respondents prior to beginning the survey and ethics approval was obtained from the Survey and Behavioural Research Ethics Committee of The Chinese University of Hong Kong [Ref No. SBRE-09-17]. Details of the data collection procedure and survey have been previously described [32].

### Measures

Background characteristics potentially associated with mental health outcomes were included (ethnicity, age, educational attainment, and years working as a MDW in Hong Kong) [32]. Self-rated health was measured using the EQ-5D visual analogue score, with a 0 (worst health) to 100 (best health) score based on one’s assessment of their overall health [60]. The score was dichotomized by those below the interquartile range (IQR) (labeled with “poor” health) and those within or above IQR (labeled with “average/good” health).

#### Perceived Discrimination

Perceived discrimination was assessed by asking “Since living in Hong Kong, have you ever felt you were being discriminated against because of your domestic helper’s status?” Responses were given on a 5-point Likert scale (1 = Never to 5 = Always) and was measured as a continuous variable to assess the trend in increasing frequencies of perceived discrimination.

#### Stress

Stress was measured using a single-item question which was validated and found to be reliable among working populations [61, 62]. The item states, “Stress refers to a situation where a person feels tense, restless, nervous, or anxious, or is unable to sleep at night because his/her mind is troubled all the time. Do you feel that kind of stress these days?” Responses were scored on a 5-point Likert scale (1 = not at all to 5 = very much) with higher scores indicating more stress. The item was assessed as a continuous variable.

#### Perceived Social Support

Perceived social support was measured using the unidimensional 6-item brief form of the Perceived Social Support Questionnaire (F-SozU K-6) [63]. F-SozU K-6 measures general situations of social support (e.g., “I receive a lot of understanding and security from others”) using a 5-point Likert scale where a mean score (range 1–5) is calculated with higher scores indicating more perceived social support. F-SozU-K6 has been found as a reliable and valid measure across several populations [63, 64], and demonstrated high internal consistency in our study sample (α = 0.88 English, 0.90 Indonesian).

#### Mental Health

Anxiety was assessed using the Generalized Anxiety Disorder 7-item scale (GAD-7) [65], and depression was measured with the Patient Health Questionnaire 9-item scale [66]. Both measures asked respondents to rate the frequency of negative feelings over the past two weeks on a 4-point Likert scale (0 = “not at all” to 3 = “nearly every day”), with higher summative scores indicating greater symptoms (ranges: GAD-7, 0–21; PHQ-9, 0–27). Both measures have been previously validated among Filipino and Indonesian MDWs [67, 68] and showed high internal reliability in our sample (GAD-7 α = 0.89 English, 0.86 Indonesian; PHQ-9 α = 0.83 English, 0.84 Indonesian). Scores were analyzed as continuous variables.

### Analysis

Descriptive statistics were calculated with percentage and frequency for all categorical variables. ANOVA was used to assess mean score differences of GAD-7 and PHQ-9 within each variable. Correlations between each variable were tested using Spearman’s rank correlation coefficient.

Moderated mediation is an analysis that uses linear regression models to test different pathways of significance. In this analysis, perceived discrimination was considered for the independent variable, stress as the mediating variable, social support as a moderating variable, and anxiety and depression as the outcomes. Mediation was tested from perceived discrimination to stress (a-path) and stress to anxiety and depression (b-path). Using Hayes’ PROCESS macro Model 15, moderation was tested on both the direct path from perceived discrimination to anxiety and depression (c-path) and on the b-path (Fig. 1) [69]. The moderated mediation effect was tested using the index of moderated mediation where bootstrapping with 5000 resamples was used to test the significance of the indirect effects. A 95% confidence interval that did not cross 0 was considered a significant moderated mediation. All continuous variables were centered using − 1 standard deviation, mean, and + 1 standard deviation for analysis, and all covariates were included to control for potential confounders. All analysis was completed using SPSS version 26 and PROCESS macro for SPSS [69, 70].

## Results

### Description of the Study Sample

The study sample (see Table 1) was mostly Filipino (81.5%), aged 35–44 (48.5%), not married (53.0%), and obtained a secondary school education (40.7%). One-third of the respondents had been working in Hong Kong as a MDW for 1–3 years while just over one-fifth reported having done so for 10 or more years. Discrimination based on domestic worker status was reported by 60.4% of respondents. Experiencing discrimination “often” or “always” was reported by 10.6% of respondents. About 37% of MDWs reported feeling no stress recently. The mean score of perceived social support was 3.55 (of 5). Details of the F-SozU-K6 scale can be found in Supplementary Table 2.

**Table 1:** Background characteristics of the study sample (*n* = 1965)

			Anxiety^c^		Depression^c^		
Variables	%	n	mean	p	mean	p	
**Perceived discrimination**							
Have you ever felt discriminated because of your MDW status in Hong Kong?				**		**	
Never	39.6%	779	1.7		2.4		
Seldom	9.8%	193	2.9		4.0		
Sometimes	40.0%	785	3.9		4.8		
Often	5.0%	99	5.4		6.1		
Always	5.6%	109	5.7		6.9		
**Stress** ^**a**^				**		**	
Not at all	37.0%	727	1.1		1.5		
Only a little	38.8%	763	3.3		4.5		
To some extent	12.6%	248	5.1		6.0		
Rather much	4.3%	84	6.3		7.4		
Very much	7.3%	143	6.8		7.9		
**Perceived social support scale (1–5)** ^**b**^				*		**	
-1SD	13.7%	269	3.1		3.8		
Mean (3.55)	68.8%	1352	3.2		4.1		
+1SD	17.6%	345	2.5		3.3		
**Covariates**							
Ethnicity				**		*	
Filipino	81.5%	1601	3.2		4.0		
Indonesian	18.5%	364	2.5		3.5		
Age				**		**	
20–34	35.2%	691	3.7		4.6		
35–44	48.5%	954	2.9		3.8		
≥ 45	16.3%	320	2.3		3.2		
Educational attainment				**		**	
Secondary school	40.7%	799	2.8		3.6		
Technical or vocational school	27.2%	535	3.3		4.1		
University or postgraduate degree	32.1%	631	3.3		4.3		
Years working as MDW in Hong Kong				**		**	
1–3	33.6%	661	3.6		4.2		
4–9	44.9%	882	3.2		4.2		
≥ 10	21.5%	422	2.3		3.1		
Self-rated health				**		**	
Poor (< IQR)	24.3%	478	5.2		6.0		
Average/good (≥ IQR)	75.7%	1487	2.4		3.3		

*p < 0.05; **p < 0.01. Interquartile range = IQR. Standard deviation = SD. ^a^ Question stated: “Stress refers to a situation where a person feels tense, restless, nervous, or anxious, or is unable to sleep at night because his/her mind is troubled all the time. Do you feel that kind of stress these days?” ^b^ Higher score indicates more perceived social support. ^c^ Anxiety assessed with GAD-7 (score range 0–21) and depression assessed with PHQ-9 (score range 0–27), where a higher score indicates more anxiety and depressive symptoms

In unadjusted linear regression, higher perceived discrimination, increased stress, lower perceived social support, Filipino ethnicity, younger age, higher education, fewer years as a MDW in Hong Kong, and lower self-rated health were each significantly associated with higher anxiety and depression scores.

### Study Variable Correlates

Supplementary Table 1 presents the correlations between the background variables and the main study variables. Perceived discrimination was significantly correlated with stress (*r* = 0.28, *p* < 0.01), anxiety (*r* = 0.37, *p* < 0.01), depression (*r* = 0.37, *p* < 0.01), as well as social support (*r* = -0.08, *p* < 0.01) and all background variables except for years as a MDW in Hong Kong.

### Moderated Mediation Analysis

The moderated mediation analysis results, including conditional direct and indirect effects of perceived discrimination on anxiety and depression, are presented in Table 2. Perceived discrimination was significantly associated with stress (a-path: b = 0.186, p < 0.001). In the anxiety model, stress and social support significantly interacted on the b-path (b = -0.332, p = 0.003, ΔR² = 0.007), with a significant index of moderated mediation (b = -0.062, 95% CI [-0.106, -0.062]). The direct effect (c’) of perceived discrimination on anxiety was not moderated by social support but remained independently significant (b = 0.622, p = < 0.001).

**Table 2:** Regression results for the a-path from perceived discrimination to stress, for the b-path from stress to anxiety and depression, and the index of moderated mediation (*n* = 1965)

	Model a-path			Model b/c’-path			Model b/c’-path		
	Stress			Anxiety			Depression		
	b	SE	p	b	SE	p	b	SE	p
Perceived discrimination	0.186	0.023	< 0.001	0.622	0.069	< 0.001	0.722	0.073	< 0.001
Stress				1.306	0.090	< 0.001	1.462	0.093	< 0.001
Perceived social support				-0.185	0.098	0.059	-0.211	0.095	0.027
Perceived discrimination * Social support				0.033	0.080	0.686	-0.009	0.105	0.912
Stress * Social support				-0.332	0.110	0.003	-0.253	0.230	0.016
**Conditional direct effect of perceived discrimination on mental health**									
**Perceived discrimination** → **mental health**				effect	SE	p	effect	SE	p
Low perceived social support				0.595	0.096	< 0.001	0.730	0.100	< 0.001
Moderate perceived social support				0.622	0.069	< 0.001	0.722	0.073	< 0.001
High perceived social support				0.648	0.094	< 0.001	0.715	0.097	< 0.001
**Conditional indirect effect of perceived discrimination on mental health**									
**Perceived discrimination** → **Stress** → **Mental health**				effect	SE	95% CI	effect	SE	95% CI
Low perceived social support				0.293	0.044	0.213, 0.385	0.310	0.046	0.227, 0.407
Moderate perceived social support				0.243	0.035	0.179, 0.313	0.272	0.039	0.202, 0.353
High perceived social support				0.193	0.032	0.136, 0.257	0.234	0.037	0.167, 0.312
**Index of moderated mediation**				Index	SE	95% CI	Index	SE	95% CI
				-0.062	0.021	-0.106, -0.023	-0.047	0.020	-0.090, -0.011

PD = perceived discrimination; SE = standard error. Models adjusted for ethnicity, age, educational attainment, years working as a MDW in Hong Kong, and self-rated health. Model for the a-path R² = 0.12, F(7, 1957) = 35.92, p < 0.001, Model for b-path and c’-path (Anxiety) R² = 0.34, F(11, 1953) = 60.04, *p* < 0.001, Model for b-path and c’-path (Depression) R² = 0.35, F(11, 1953) = 64.541, *p* < 0.001

In the depression model, a similar interaction between stress and social support was observed on the b-path (b = -0.253, p = 0.016, ΔR² = 0.004), with a significant index of moderated mediation (b = -0.047, 95% CI [-0.090, -0.011]). The direct effect (c’) of perceived discrimination on depression was also not moderated by social support but was independently significant (b = 0.722, p = < 0.001).

Both models provided evidence of moderated mediation, with the largest buffering effect of perceived social support on stress observed at the highest stress levels. Supplementary Fig. 1 illustrates this moderation effect. All results were controlled for by the covariates previously stated. All final models reported a low VIF range (1.03 to 1.88).

## Discussion

### New Contribution to the Literature

This study highlights significant associations between perceived discrimination, stress, perceived social support, and mental health outcomes among MDWs in Hong Kong. Higher frequencies of perceived discrimination were directly associated with anxiety and depression symptoms. Stress partially mediated this association, while perceived social support significantly buffered stress’s effect on anxiety and depression but not the direct effect of perceived discrimination and mental health. These findings demonstrate the need for a combination of short-term targeted interventions and long-term policy change to reduce discrimination, alleviate stress, and enhance social support among MDWs.

### Discrimination Against MDWs

Study participants reported perceived discrimination based on their employment status, but the intersectionality of MDWs – as female, ethnic minority, migrant workers with lower social status, and distinct religious identities, particularly hijabi MDWs who are readily identifiable by their employers and others – likely exposed them to multiple additional forms of discrimination. Existing ordinances in Hong Kong address discrimination based on sex, family status, disability, and race [71], but MDWs’ reliance on their employers for legal working status may hinder reporting of direct or indirect discrimination for fear of job loss [72]. Simplifying complaint procedures and ensuring confidentiality are crucial to encourage reporting and address issues of discrimination.

Workplace discrimination, reported by MDWs globally, is perceived through systemic power imbalances, dehumanization, limited autonomy, and job immobility [25, 30]. These were often heightened during the COVID-19 pandemic [28, 73–78]. In regions such as Hong Kong and the Middle East, discriminatory language from employers and media further reveals structural inequities undermining MDWs’ rights and dignity [27, 52, 79–82]. These patterns highlight the urgent need for policy reforms to create more equality by addressing regulations that disproportionately favor employers. In consideration of MDWs’ human rights, such policies can better protect MDWs from discrimination, promote their autonomy, and affirm their contributions to the global workforce.

### Long-Term Policy Implications

To address issues of workplace discrimination for MDWs, policies first need to improve MDW autonomy and their ability to escape intolerable, and at times, illegal treatment. Current working visa schemes, enforced in Hong Kong, the Middle East, and Singapore [20, 21, 83], require employer sponsorship, making MDWs’ continued ability to work highly dependent on their good standing with their employers. Also, requirements to live with their employer and to repatriate very shortly after their contract ends further diminish MDW autonomy if they wish to cease working for their employer. Therefore, decoupling MDW working visas from individual employer’s sponsorship and extending the time to find new employment would reduce the power imbalance between MDWs with their employer. Additionally, permitting MDWs to reside outside their place of employment may also reduce stress from their workplace and provide opportunities for building stronger support networks with other MDWs.

In addition, policies to improve MDWs’ ability to seek alternative employment, should also include enforcement of existing regulations that stipulate minimum standards for living accommodations and safe working environments. While routine inspection of employment spaces would be theoretically ideal, they are infeasible due to requirement of warrants for home inspections. Alternatively, the implementation of a confidential complaint hotline to report violations could help ensure employer compliance with existing regulations. Since policy changes require considerable political will to address legislatively, more immediate interventions are needed to address mental health needs of MDWs.

### Interventions to Improve Mental Health and Reduce Stress in MDWs

To address the poor mental health and high levels of stress reported by MDWs, several short-term actions can also be taken. Since MDWs face time constraints for seeking traditional forms of mental health services and may face employer disapproval or even termination of their contracts, mental health interventions must be discreet, low-cost and readily accessible. Digital mental health interventions, such as the WHO’s Step-by-Step phone-based app [84], could partially address the mental health challenges faced by MDWs. Prior research shows positive responses among Filipino MDWs in Macao when using digital mental health tools and the Step-by-Step app has undergone cultural adaptation and evaluation to assess its effectiveness among this population [85–88]. These types of guided, technology-supported interventions could thereby address treatment gaps in MDW populations [84].

Recent research confirms the significant buffering effect of perceived social support on stress, emphasizing its importance for MDWs’ well-being [44]. The COVID-19 pandemic, which increased social isolation for many MDWs [25, 78, 89] has further emphasized the urgency of rebuilding these networks. MDWs frequently rely on informal networks, such as family, friends, and religious institutions for social and emotional support [46, 90]. However, systemic barriers, including demanding work schedules and limited autonomy, may restrict their ability to maintain or rebuild these connections. Broader policy reforms are essential to provide MDWs with the time and agency required to engage with these networks effectively. Moreover, local governments could provide additional financial resources to community organizations, NGOs, and religious institutions already serving and providing support to MDWs. By addressing both immediate resource gaps and structural barriers, governments can help MDWs rebuild social support networks.

### Limitations and Future Research

Limitations include the cross-sectional design which precludes causal inferences and non-probability sampling which potentially limited representativeness; though study sample age distributions were comparable to population data. Perceived discrimination was measured with a single non-validated item, though it showed expected association with mental health outcomes. Self-reported measures risk recall and social desirability biases, although shorter recall periods and anonymity should have reduced these biases. The study survey was offered in English and Indonesian, potentially excluding Filipinos more fluent in Tagalog than English. However, during survey pilot testing, Filipinos overwhelmingly preferred the English version to Tagalog, and this preference was found in another study of Filipino MDWs [91]. Seeking key informants’ perspectives through interviewing in the research design phase can help to avoid such limitations in the future.

Future research could adopt a longitudinal design to establish causal inference between perceived discrimination and mental health status. Moreover, integrating multiple methodologies to measure discrimination, such as qualitative narrative and standardized scales, would provide a more comprehensive understanding of this multifaceted social construct, as discrimination may also be based on gender, religious identity, and/or migrant status. Future research may also assess the perceived feasibility of changes to MDW policy among key stakeholders (i.e., employers, policymakers, employment agencies).

## Conclusion

In this study, perceived discrimination based on employment status was a frequently reported occurrence among MDWs and had both direct and indirect effects on their mental health. Amendments to policies which may exacerbate and perpetuate discriminatory behavior, including unlimited working hours and live-in requirements, should be considered. In the interim, stress and perceived social support may provide targetable factors to address the negative mental health effects of perceived discrimination. Interventions including app-based mental health services and building social support networks may help improve MDW well-being.

## Electronic supplementary material

Below is the link to the electronic supplementary material.

Supplementary Material 1

Supplementary Material 2

Supplementary Material 3

## Data Availability

Data is available upon reasonable request to the corresponding author.
