# Supplementary material for: The Role of Stress and Perceived Social Support in the Association Between Perceived Discrimination and Mental Health Among Migrant Domestic Workers in Hong Kong
Source: J Immigr Minor Health. 2025 May 20;27(5):677–86. doi: 10.1007/s10903-025-01694-x (PMC12420704; doi:10.1007/s10903-025-01694-x)
Supplement: Supplementary file 1 — Supplementary Material 1 [file 10903_2025_1694_MOESM1_ESM.docx]

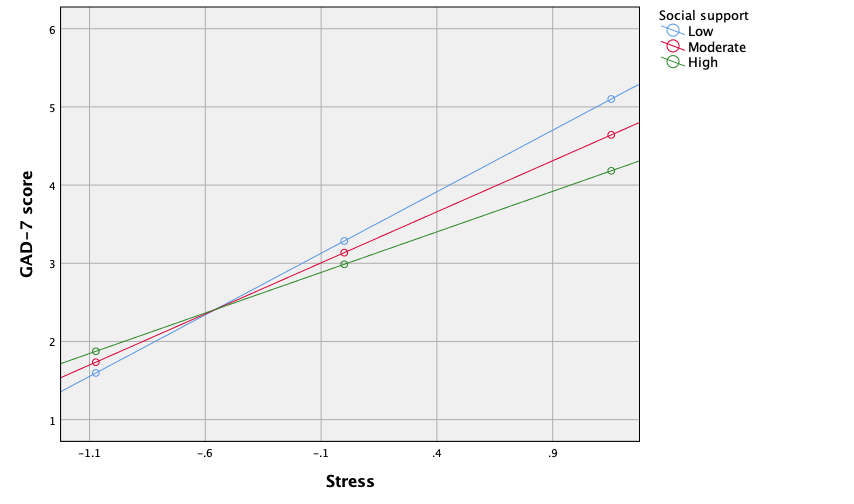


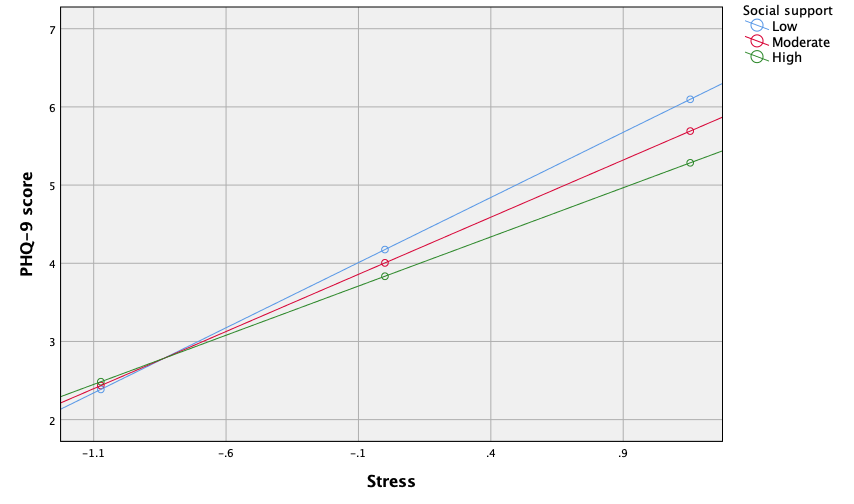


**Supplementary Figure 1.** Moderation effect of perceived social support on stress and anxiety and depression

GAD-7 = Generalized Anxiety Disorder-7 scale (Anxiety); PHQ-9 = Patient Health Questionnaire-9 (Depression). Three points on the X-axis indicate -1 SD, mean, and +1 SD. Social support categories = low (-1SD), moderate (mean), and high (+1 SD)
