# Supplementary material for: The Role of Stress and Perceived Social Support in the Association Between Perceived Discrimination and Mental Health Among Migrant Domestic Workers in Hong Kong
Source: J Immigr Minor Health. 2025 May 20;27(5):677–86. doi: 10.1007/s10903-025-01694-x (PMC12420704; doi:10.1007/s10903-025-01694-x)
Supplement: Supplementary file 2 — Supplementary Material 2 [file 10903_2025_1694_MOESM2_ESM.docx]

**Supplementary Table 1.** Bivariate correlations among the study variables (n=1965)

|  | 1 | 2 | 3 | 4 | 5 | 6 | 7 | 8 | 9 | 10 |
| --- | --- | --- | --- | --- | --- | --- | --- | --- | --- | --- |
| 1 Ethnicity | -- |  |  |  |  |  |  |  |  |  |
| 2 Age | 0.12** | -- |  |  |  |  |  |  |  |  |
| 3 Educational attainment | -0.33** | -0.04 | -- |  |  |  |  |  |  |  |
| 4 Years working in HK as an MDW | 0.22** | 0.47** | 0.02 | -- |  |  |  |  |  |  |
| 5 Self-rated health | -0.02 | 0.01 | -0.00 | -0.02 | -- |  |  |  |  |  |
| 6 Perceived discrimination | -0.22** | -0.07** | 0.19** | -0.04 | -0.17** | -- |  |  |  |  |
| 7 Stress | -0.14** | -0.08** | 0.11** | -0.06** | 0.26** | 0.28** | -- |  |  |  |
| 8 Perceived social support scale | -0.21** | -0.05* | 0.12** | -0.04 | -0.12** | -0.08** | -0.01 | -- |  |  |
| 9 Anxiety | -0.11** | -0.15** | 0.08** | -0.14** | 0.31** | 0.37** | 0.53** | -0.08** | -- |  |
| 10 Depression | -0.08** | -0.15** | 0.10** | -0.11** | 0.29** | 0.37** | 0.56** | -0.08** | 0.76** | -- |

*p<0.05; **p<0.01
