# Supplementary material for: The Role of Stress and Perceived Social Support in the Association Between Perceived Discrimination and Mental Health Among Migrant Domestic Workers in Hong Kong
Source: J Immigr Minor Health. 2025 May 20;27(5):677–86. doi: 10.1007/s10903-025-01694-x (PMC12420704; doi:10.1007/s10903-025-01694-x)
Supplement: Supplementary file 3 — Supplementary Material 3 [file 10903_2025_1694_MOESM3_ESM.docx]

**Supplementary Table 2**. Characteristics of the Brief Perceived Social Support Questionnaire (n=1965)

|  | Total | | Not true at all | | Somewhat not true | | Neutral | | Somewhat true | | Very true | |
| --- | --- | --- | --- | --- | --- | --- | --- | --- | --- | --- | --- | --- |
| Item | Mean | SD | % | n | % | n | % | n | % | n | % | n |
| 1. I receive a lot of understanding and security from others. | 3.23 | 1.08 | 9.2% | 180 | 6.8% | 134 | 50.3% | 988 | 19.1% | 376 | 14.6% | 287 |
| 2. There is someone very close to me whose help I can always count on. | 3.73 | 1.11 | 4.6% | 90 | 5.2% | 102 | 35.4% | 696 | 22.1% | 435 | 32.7% | 642 |
| 3. If I need to, I can borrow something from friends or neighbors without any problems. | 3.47 | 1.16 | 7.9% | 155 | 7.5% | 147 | 38.5% | 757 | 22.2% | 437 | 23.9% | 469 |
| 4. I know several people with whom I like to do things. | 3.57 | 1.05 | 4.9% | 97 | 4.1% | 81 | 43.1% | 847 | 24.3% | 477 | 23.6% | 463 |
| 5. When I am sick, I can ask friends / relatives to handle important things for me without hesitation. | 3.58 | 1.18 | 7.7% | 151 | 6.0% | 118 | 35.5% | 698 | 22.1% | 434 | 28.7% | 564 |
| 6. If I’m very depressed, I know who I can turn to. | 3.77 | 1.15 | 6.4% | 126 | 3.1% | 61 | 32.3% | 633 | 23.7% | 466 | 34.6% | 679 |
| **Total** | 3.56 | 0.81 |  |  |  |  |  |  |  |  |  |  |

Item scores range from 1 (Not true at all) to 5 (Very true). Total score is the sum of each item’s mean score divided by 6.
